# One way coupling of CMAQ and a road source dispersion model for fine scale air pollution predictions

**DOI:** 10.1016/j.atmosenv.2012.05.034

**Published:** 2012-11

**Authors:** Sean D. Beevers, Nutthida Kitwiroon, Martin L. Williams, David C. Carslaw

**Affiliations:** MRC-HPA Centre for Environment and Health, Kings' College London, London, United Kingdom

**Keywords:** Road source modelling, CMAQ, Air quality, Model evaluation, London

## Abstract

In this paper we have coupled the CMAQ and ADMS air quality models to predict hourly concentrations of NO_*X*_, NO_2_ and O_3_ for London at a spatial scale of 20 m × 20 m. Model evaluation has demonstrated reasonable agreement with measurements from 80 monitoring sites in London. For NO_2_ the model evaluation statistics gave 73% of the hourly concentrations within a factor of two of observations, a mean bias of −4.7 ppb and normalised mean bias of −0.17, a RMSE value of 17.7 and an *r* value of 0.58. The equivalent results for O_3_ were 61% (FAC2), 2.8 ppb (MB), 0.15 (NMB), 12.1 (RMSE) and 0.64 (*r*). Analysis of the errors in the model predictions by hour of the week showed the need for improvements in predicting the magnitude of road transport related NO_*X*_ emissions as well as the hourly emissions scaling in the model. These findings are consistent with recent evidence of UK road transport NO_*X*_ emissions, reported elsewhere. The predictions of wind speed using the WRF model also influenced the model results and contributed to the daytime over prediction of NO_*X*_ concentrations at the central London background site at Kensington and Chelsea. An investigation of the use of a simple NO–NO_2_–O_3_ chemistry scheme showed good performance close to road sources, and this is also consistent with previous studies. The coupling of the two models raises an issue of emissions double counting. Here, we have put forward a pragmatic solution to this problem with the result that a median double counting error of 0.42% exists across 39 roadside sites in London. Finally, whilst the model can be improved, the current results show promise and demonstrate that the use of a combination of regional scale and local scale models can provide a practical modelling tool for policy development at intergovernmental, national and local authority level, as well as for use in epidemiological studies.

## Highlights

► We coupled the CMAQ and ADMS air quality models. ► We predicted hourly NO_*X*_, NO_2_ and O_3_ in London at 20 m × 20 m. ► Model evaluation has demonstrated reasonable agreement with 80 monitoring sites in London. ► Improvements are required in predicting the magnitude of road transport NO_*X*_ emissions. ► The combination of regional and local scale models is a practical modelling tool.

## 1 Introduction

Air quality models are important tools in providing evidence of the impact on air quality of policy options at both regional and national scale under obligations to the National Emission Ceilings Directive (NEDC, 2001) and the Gothenburg Protocol (UNECE, 1999), and at city scale, to implement plans such as the Mayors Air Quality Strategy (GLA, 2010b) in London and numerous Low Emission Zones across Europe (http://www.lowemissionzones.eu/). In the UK concerns are focused on meeting NO_2_, PM_10_ and PM_2.5_ EU limit values (EC, 2008), on the impacts of short term climate forcing and on the impacts on sensitive ecosystems and crops. However, increasingly models are also being used as a source of human exposure information for epidemiological studies (Maheswaran et al., 2010; Tonne et al., 2010) and health impact assessments (e.g., Georgopoulos et al., 2005; Isakov et al., 2009; Woodcock et al., 2009). Epidemiological studies, which look cross-sectionally at health outcomes, require models to provide annual mean concentrations at fine spatial scales (post/zip code areas), but are increasingly required to elucidate a combination of spatio-temporal influences placing further demands on a model's temporal resolution. Finally, there is a need to take account of space-time-activity data (Ashmore, 2005) in exposure assessments both for policy development and to reflect the dose from pollutants that have a range of toxicity (such as PM) (Kelly et al., 2011) or of mixtures of pollutants (Molitor et al., 2011).

In an attempt to meet these demands in a large urban area, we describe the coupling of the Community Multiscale Air Quality (CMAQ) modelling system (Byun and Ching, 1999) and the Atmospheric Dispersion Modelling System (ADMS) Roads model (CERC, 2006). We provide details of the models performance in predicting hourly NO_*X*_–NO_2_–O_3_ concentrations over a detailed (20 m × 20 m) grid in London in 2006. The results for a similar run in 2008 are also provided in support of the 2006 evaluation and have been reported elsewhere (Carslaw, 2011).

## 2 Methods

### 2.1 CMAQ-urban COULPING

The regional scale (CMAQ) and local scale (ADMS) dispersion models have been coupled to create a model system (CMAQ-urban). In Section 2.2 details are given of the Weather Research and Forecasting (WRF) setup, in Section 2.3 details are given of the CMAQ setup, in Section 2.4 details are given of the ADMS setup and in Section 2.5 details are given of the outputs of CMAQ-urban.

### 2.2 WRF setup details

The WRF model v3.1 (Skamrock et al., 2008) was setup using 23 vertical layers with 7 layers under 800 m and the top layer ∼15 km above the ground. Four horizontal nesting levels were used, downscaling from 81 km grids over Europe to 27 km over the UK and Ireland, 9 km over the UK and 3 km over London. Initial and boundary (IC/BC) conditions were taken from the GFS model at 1 × 1° (http://www.esrl.noaa.gov/psd/data/reanalysis/reanalysis.shtml). Other physics settings within WRF included use of the Rapid Radiation Transfer Model (RRTM) radiation scheme (Mlawer et al., 1997), the Kain–Fritsch (new Eta) microphysics scheme (Kain, 2004), the Yonsei University (YSU) planetary boundary layer scheme (Hong et al., 2006), the Monin–Obukhov surface scheme (Skamrock et al., 2008) and the National Ocean and Atmospheric Administration (NOAA) land surface scheme (Chen and Dudhia, 2001). Observational nudging of wind speed, temperature and relative humidity was used in the WRF model with a nudging strength of 6 × 10^−4^ and radius of influence of 200 km. In urban areas urban land cover and *Z*_0_ values of 0.8 m were used. No anthropogenic heat from buildings or road transport was included in the WRF model.

### 2.3 CMAQ setup details

The CMAQ v4.6 model was setup using the same vertical and horizontal grid structure as for WRF. Atmospheric chemistry was simulated using the CB 05 chemical mechanism (Yarwood et al., 2005) and initial and boundary conditions, included as monthly means, from the UK Meteorological Office chemistry-transport model, STOCHEM (Derwent pers. comm.), which provided 14 pollutant species including CO, NH_3_, NO_*X*_, O_3_, HNO_3_ and VOC's. Anthropogenic and natural emissions were derived from the European Monitoring and Evaluation Programme (EMEP, http://www.ceip.at/), the European Pollutant Release and Transfer Register (EPETR), the National Atmospheric Emissions Inventory (NAEI) (Murrells et al., 2010) and the London Atmospheric Emissions Inventory (LAEI) (GLA, 2010a). The emissions were summarised into 11 Selected Nomenclature for sources of Air Pollution or SNAP sectors and were processed into gridded chemical species using speciation and temporal profiles developed in the US–EU project, Air Quality Modelling Evaluation International Initiative (AQMEII) (http://aqmeii.jrc.ec.europa.eu/). Hourly emissions for road traffic were created using a time series of normalised traffic counts, taken from 37 automatic sites in London. The monthly and hour of week profiles for each SNAP sector were processed using the Sparse Matrix Operator Kernel Emissions model (SMOKE v 2.4). Biogenic emissions were estimated using high resolution Coordination of Information on the Environment (CORINE) land cover data (http://www.eea.europa.eu/data-and-maps), meteorological data from WRF and methods described by Guenther et al. (1995) and Sanderson (2002).

The input emissions data were provided at a range of spatial scales, from 50 km grids across Europe (EMEP) down to 1 km grids for the NAEI and LAEI. The link between each emissions source and the CMAQ model domains was undertaken by re-projecting all of the emissions data into the Lambert Conformal projection and then intersecting each emissions database and model domain in ArcGIS v9.3. Where a CMAQ grid cut an emissions grid into two or more pieces, a proportion of the emissions was added to the model grid, based upon the intersected area.

Road transport NO and exhaust primary NO_2_ emissions for the UK and London, were taken from the NAEI and the LAEI, respectively, and released into the lowest layer of the model, 14 m high. Assumptions for exhaust primary NO_2_ emissions were taken from Grice et al. (2009). Large industrial plant emissions from ∼10,000 sources in the EU were taken from the NAEI and EPETR databases and included major power stations, iron and steel smelting and oil refineries. For 100 UK sources, details of stack heights, stack diameters, volume flow rates and release temperatures were used by SMOKE to account for the effects of plume rise. In London, commercial and domestic gas combustion for space heating, which represents a large source of NO_*X*_ (GLA, 2010a), and all other ground based sources of NO_*X*_ were released into the lowest 14 m layer of the CMAQ model.

### 2.4 ADMS setup details

The ADMS roads model (v2.3) (CERC, 2006) was used to describe the near field dispersion from roadways in CMAQ-urban, using the hourly meteorological inputs: wind speed and direction, temperature, surface sensible heat flux and planetary boundary layer height, predicted from the WRF model. The ADMS model was run for each hour of the year using similar methods to those described in Kelly et al. (2011), to produce hourly concentration fields or model kernels. Each kernel represents the concentration of a primary pollutant (with no chemistry applied) across a regular grid as it dilutes away from a road source of unit emissions. The concentrations across each kernel were predicted at 5 m intervals and within 225 m of each source, using a constant road emissions rate of 1 g km^−1^ s^−1^. Road geography is highly important and so the emissions from roads in London were represented using the centreline of each carriageway divided into 10 m sections. The 10 m granularity of the road sources was considered to be representative of complex road curves but also the distance between carriageways on larger roads such as motorways. Six road categories (and associated kernels) were used in London, including open roads (motorway), typical roads (average urban roads surrounded by low rise buildings) and 4 types of street canyon (classified by their orientation: north–south, east–west, southwest–northeast and southeast–northwest). The “typical roads” had a road width of 20 m and a building height of 10 m and “street canyons” had a width of 30 m and a building height of 25 m. The “street canyon” width and height details were manually sampled from the 3D building model in London (http://www.casa.ucl.ac.uk/news/newsStory.asp?ID=80). Once created, each kernel was applied to the 1746 major road emissions estimates in the LAEI consisting of ∼340,000 10 m road sources, and the hourly concentration of NO and primary exhaust NO_2_ combined onto a fixed grid of 20 m × 20 m in London.

The near road chemistry was simulated using a simple chemical scheme described in Carslaw and Beevers (2005). The reaction rates and photo-dissociation rates were taken from the photolysis rate pre-processor (JPROC) (Roselle et al., 1999), part of the CMAQ run, and the time of flight from road sources, estimated each hour, as a concentration weighted average at each receptor location, assuming a straight line between source and receptor and using WRF wind speed at 10 m height.

### 2.5 CMAQ-urban outputs

The CMAQ predictions of hourly NO, NO_2_ and O_3_ at 3 × 3 km in London were downscaled to a fixed 20 m × 20 m grid using the bilinear interpolation method described in Shao et al. (2007). The CMAQ hourly concentrations are smoothly varying in space for each hour and this limits the error introduced through the interpolation step and allows a direct match with the ADMS model outputs. The ADMS predicted concentrations of NO and primary exhaust NO_2_ were then added to the CMAQ NO, NO_2_ and O_3_ concentrations, over the fixed 20 m grid, and the simple chemistry scheme applied to give 87.2 billion hourly predictions of NO, NO_2_ and O_3_ across London in 2006. The model domain was bounded by the M25 orbital motorway.

The results from CMAQ-urban model were then analysed using methods described in the UK Department of the Environment, Food and Rural Affairs (DEFRA) model evaluation protocol (AEA, 2009) and reported using the ‘OpenAir’ software (Carslaw and Ropkins, 2012). In the model evaluation, use was made of 80 monitoring stations from the UK and London monitoring networks (http://www.londonair.org.uk/LondonAir/Default.aspx), including 22 urban background, 13 suburban, 38 roadside and 7 kerbside sites. Statistical measures of model performance (Chang and Hanna, 2004 and Builtjes, 2005) included the fraction within factor of two of the observations (FAC2), the mean bias (MB) and normalized mean bias (NMB), the root mean square error (RMSE) and the correlation coefficient (*r*). Diagnostic evaluation used modelled and measured concentrations, averaged by hour of the week and month of the year.

## 3 NO_*X*_–NO_2_–O_3_ results

**Fig. 1:**
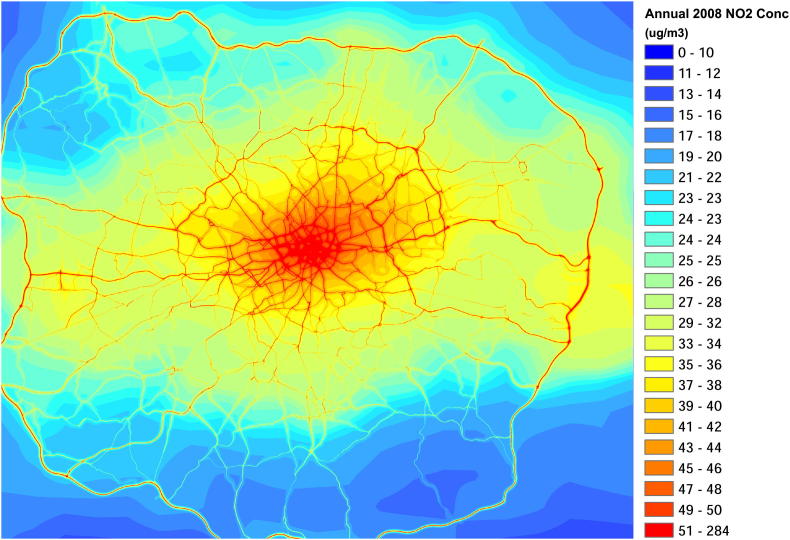
Annual 2008 mean NO_2_ concentrations for London (μg m^−3^).

The predicted 2008 annual mean NO_2_ concentrations in London are shown in Fig. 1. These results show the heterogeneity of air pollution, with areas close to major roads visible as NO_2_ concentrations of greater than ∼40 μg m^−3^. NO_2_ (and NO_*X*_) concentrations also decrease according to distance from central London, where the highest concentrations occur and at suburban background locations concentrations of NO_2_ are ∼15 μg m^−3^. As expected, the spatial distribution of O_3_ concentrations (not shown) show the opposite of the NO_2_ concentration pattern, with the minimum concentration being in the central urban area and close to road sources.

**Fig. 2:**
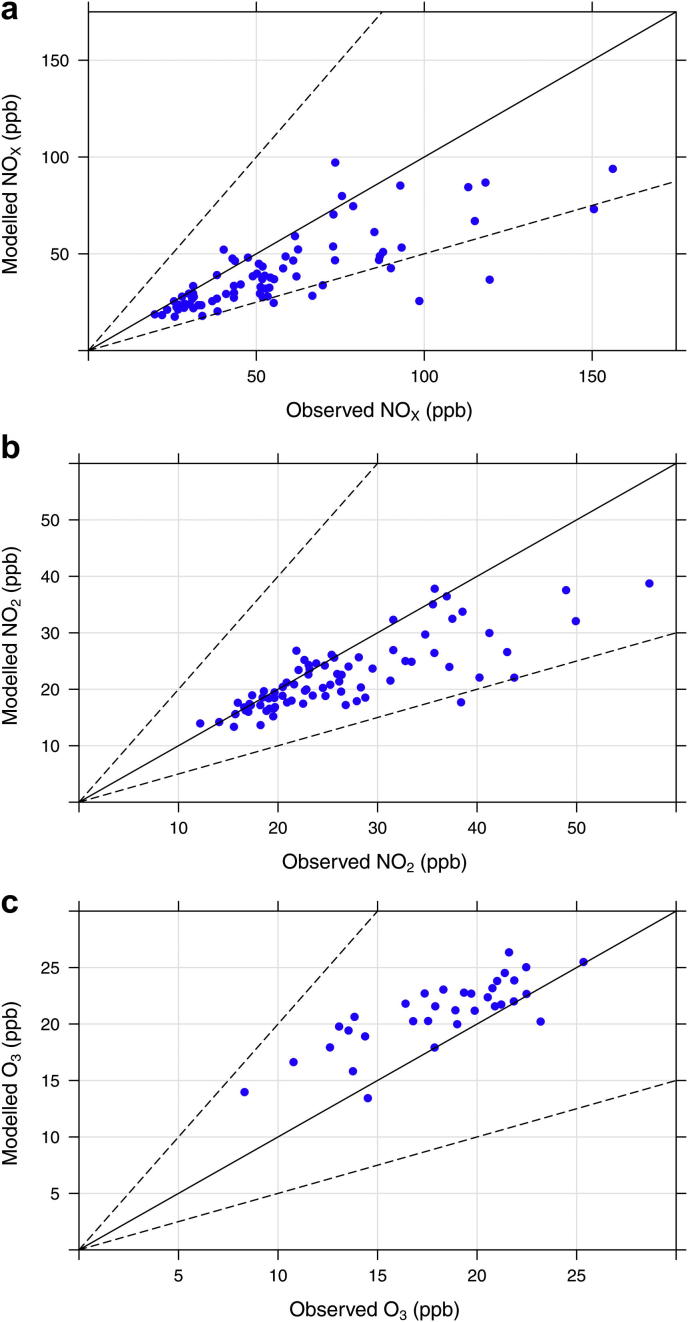
Scatter plots of annual mean NO_*X*_ (a), NO_2_ (b) and O_3_ (c) concentrations (ppb) in London in 2006. The black line represents the 1:1 relationship and the dashed lines the 1:2 and 1:0.5 relationships.

The annual mean scatter plots of NO_*X*_, NO_2_ and O_3_ are given in Fig. 2 and include a 1:1 solid black line and 1:0.5 and 1:2 dashed lines. The scatter plots in Fig. 2 show that whilst at background locations the model performs reasonably well, under predicting NO_*X*_ and NO_2_ by ∼22% and 9%, respectively, the roadside and kerbside sites under predict observations by a larger margin of ∼32% and 22%, respectively. As expected, the opposite is true for O_3_ where there is an over prediction by the model, especially close to roads of ∼18%.

**Fig. 3:**
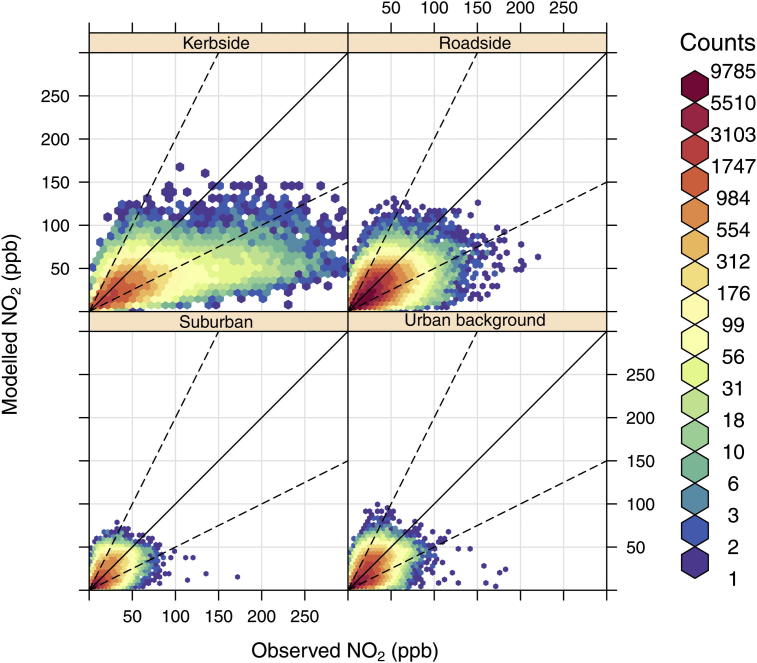
Scatter plots of hourly observed and modelled NO_2_ concentrations (ppb), at kerbside, roadside, urban background and suburban sites in 2006. The colour scale gives the hourly frequency within each scatter plot to allow clearer interpretation of the results. The black line represents the 1:1 relationship and the dashed lines the 1:2 and 1:0.5 relationships. (For interpretation of the references to colour in this figure legend, the reader is referred to the web version of this article.)

**Fig. 4:**
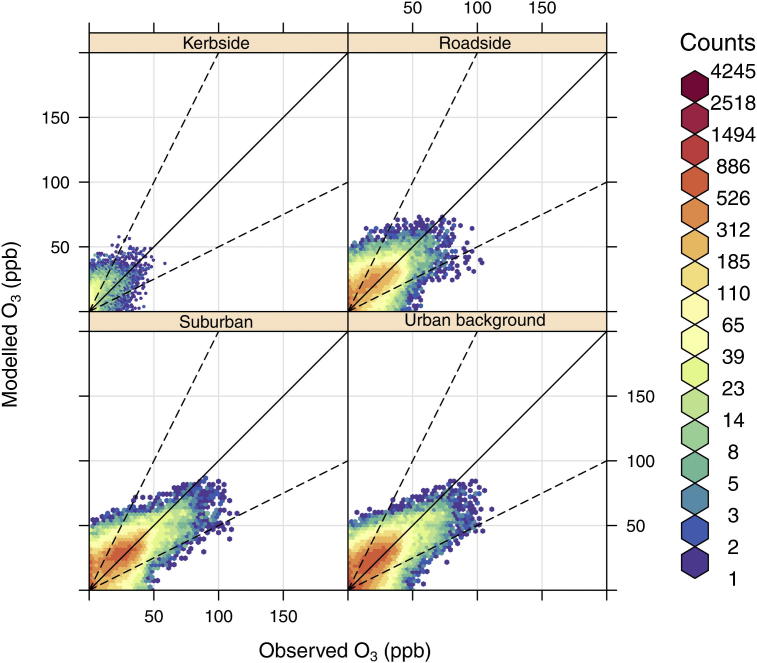
Scatter plots of observed and modelled O_3_ concentrations (ppb), at kerbside, roadside, urban background and suburban sites in 2006. The colour scale gives the hourly frequency within each scatter plot to allow clearer interpretation of the results. The black line represents the 1:1 relationship and the dashed lines the 1:2 and 1:0.5 relationships. (For interpretation of the references to colour in this figure legend, the reader is referred to the web version of this article.)

Scatter plots of the model vs. observed hourly NO_2_ and O_3_ concentrations, split into kerbside, roadside, suburban and urban background measurement site locations are shown in Figs. 3 and 4. Because it is difficult to distinguish the relationship in a scatter plot that includes thousands of points and uses only one colour, we have included a red to blue scale which reflects the number of hourly model–measurement pairs. The Figs. 3 and 4 show that the highest density of data (coloured red) is within ±FAC2 (dashed lines) for both NO_2_ and O_3_ and the results appear to show that the model predicts NO_2_ and O_3_ reasonably well at most site locations. However, a large negative bias in NO_2_ concentrations is observed at kerbside sites, especially at high concentrations, and a positive bias of O_3_ concentrations exists across all site types.

A summary of statistical measures of model performance, associated with Figs. 3 and 4, are shown in Table 1. FAC2 values indicate that for the ‘All sites’ category, more than 70% of modelled NO_2_ concentrations and 60% of modelled O_3_ concentrations are within factor of two of the observations. On average the model under predicts NO_2_ with a mean bias (MB) of approximately 5 ppb and a normalised mean bias (NMB) of −17%. The root mean squared error (RSME) and *R* value for NO_2_ are ∼18% and 0.6, respectively. In contrast, O_3_ is over predicted by approximately 3ppb giving a NMB of 15%, a RMSE of 12% and an *r* value of 0.64.

Overall the model vs. measured results, across the range of site categories, show that the model performance deteriorates as you move from background to roadside locations, with the kerbside sites showing the poorest performance of any site type. The evaluation of NO_2_ predictions at kerbside sites give the FAC2, MB, NMB, RMSE and *r* results of 0.64, −19 ppb, 0.39, 38% and 0.58, respectively, and represents an important reduction in performance, even when compared to roadside sites. Likewise the O_3_ results at kerbside locations are in poor agreement with observations.

## 4 Diagnostic evaluation of the CMAQ-urban model

Further diagnostic evaluation of the model has been used to establish the reasons for the model's performance in London. The diagnostic evaluation has focused on four areas: Hour of the week and month of the year temporal variations of NO_*X*_, NO_2_ and O_3_; comparison of the predicted and observed NO_*X*_, NO_2_ and O_3_ relationship at the Marylebone Road kerbside site; the impact of WRF wind speed predictions on modelled NO_*X*_, NO_2_ and O_3_ concentrations and the likely influence of the road traffic emissions used in the model.

### 4.1 Comparison of modelled and observed hourly NO_*X*_ at Marylebone road

**Fig. 5:**
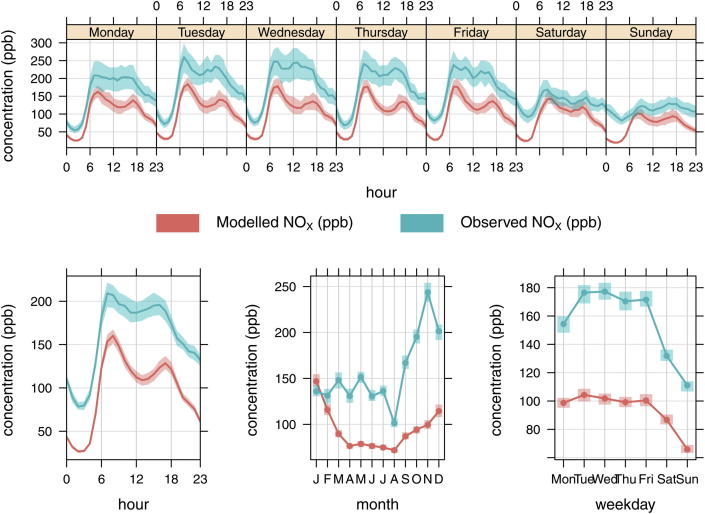
Observed and modelled NO_*X*_ (ppb) at the Marylebone road kerbside site, for hour of the day, day of the week and month of the year. The shaded area represents the 95% confidence interval in the mean.

Hour of the week and month of the year modelled and observed NO_*X*_ concentrations are plotted in Fig. 5 and show a negative bias across all periods of the week (top row plot) and a reasonable hourly emissions profile (bottom left plot). The consistent under prediction of NO_*X*_ suggests that it is at least partly associated with an under prediction of road traffic NO_*X*_ emissions. There is also evidence that the assumed reduction in emissions at weekends, compared with weekdays, is underestimated and does not compare well with measurements (bottom row right hand plot). Finally, the monthly bias increases during the period between September and December (bottom row middle plot) and this requires further investigation.

Evidence from the model results at Marylebone Road which suggest that road traffic NO_*X*_ emissions are underestimated is supported by work undertaken by Carslaw et al. (2011a, 2011b) who showed that for 10 long running roadside NO_*X*_ measurement sites in London, the period after 2004 had a median downward trend of between 0.6 and 1.7%/year and was associated with little or no improvement in the NO_*X*_ emissions performance of light duty diesel vehicles. By comparing the emissions in London over the same period, Beevers et al. (2012) has shown the downward trend in NO_*X*_ emissions for road traffic to be too large resulting in the under prediction of NO_*X*_ emissions of ∼31% by 2008. This would suggest that NO_*X*_ emissions in London (and the UK) are under predicted in 2006 by a large margin.

### 4.2 The effect of predictions of WRF wind speed on CMAQ-urban concentrations

**Fig. 6:**
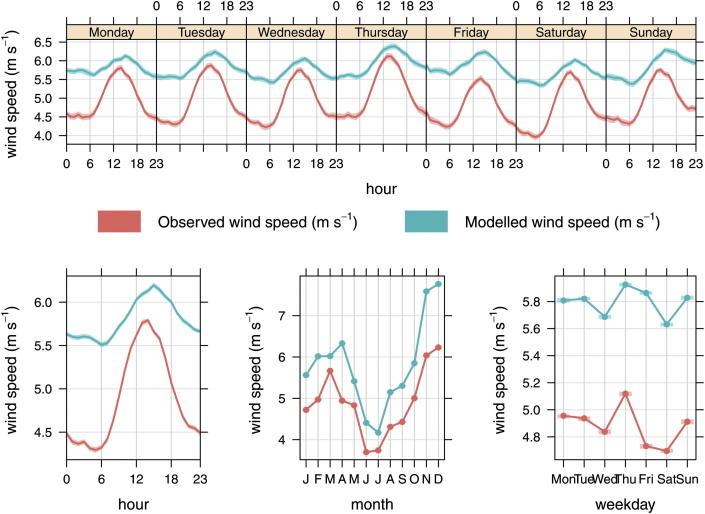
Average WRF modelled (with observational nudging) and measured wind speeds (m s^−1^) for 147 ground based meteorological stations across the UK. The shaded area represents the 95% confidence interval in the mean.

Modelled and observed wind speeds at 10 m height were compared at 147 ground based UK meteorological office stations (http://badc.nerc.ac.uk) summarised in Fig. 6. The sites are predominantly in rural locations, 103 of the 147, with 14 in urban locations and the rest coastal sites. The trend plots by hour of the week show that despite the use of observational nudging, there remains a positive bias in WRF predictions of wind speed compared with observations (top row plot). This is consistent with WRF predictions without nudging, which are not shown. The positive wind speed bias is small (∼9%) during the day, but during the overnight and morning periods the positive wind speed bias increases. Monthly comparisons also show that the bias increases slightly during winter months compared with the summer period (bottom row middle plot). Whilst, the results suggest that the model does not reflect the wind speed during overnight periods well, the nudging of temperature results in good agreement across all hours of week and months of the year.

**Fig. 7:**
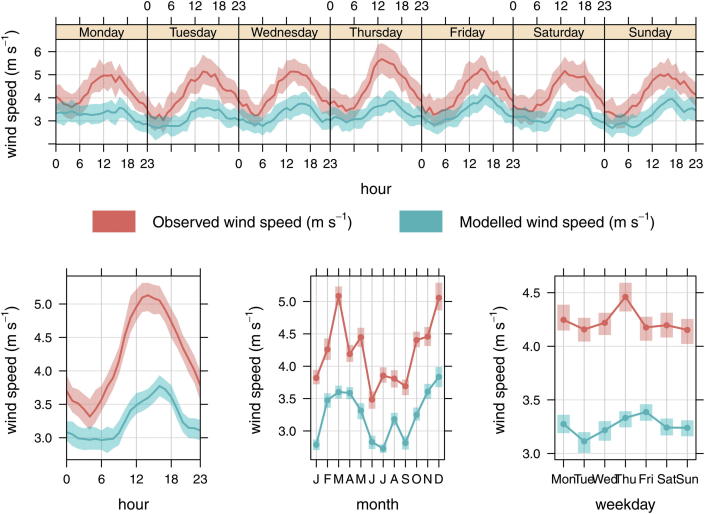
Average WRF modelled and measured wind speeds at the Heathrow Airport ground based meteorological station close to London. The model includes observational nudging. The shaded area represents the 95% confidence interval in the mean.

However, the performance of WRF at the London Heathrow site is different, and given that results at this site are likely to be more useful in interpreting the performance of CMAQ-urban we have separated out the WRF wind speed predictions at Heathrow and summarised them in Fig. 7. At Heathrow, using a combination of WRF and observational nudging has the effect of reducing the positive bias during the overnight period seen in Fig. 6, but causes the model to under predict the daytime wind speed by approximately 1.5 m s^−1^ (Fig. 7 – bottom row left plot). This is repeated at other UK urban sites. A reasonably consistent negative bias appears to exist by month of the year (bottom row middle plot) and by day of the week (bottom row right plot). The prediction of temperature at 2 m agrees with measurements at Heathrow for all hours of the week, with residuals (modelled–observed) being <0.5 °C, and reaching ∼1 °C during summer months.

The improvements in temperature predictions using observational nudging is reflected in increases in boundary layer heights compared with the without nudging case. However, a lack of observations of boundary layer height limits any conclusions that can be drawn about WRF's performance.

**Fig. 8:**
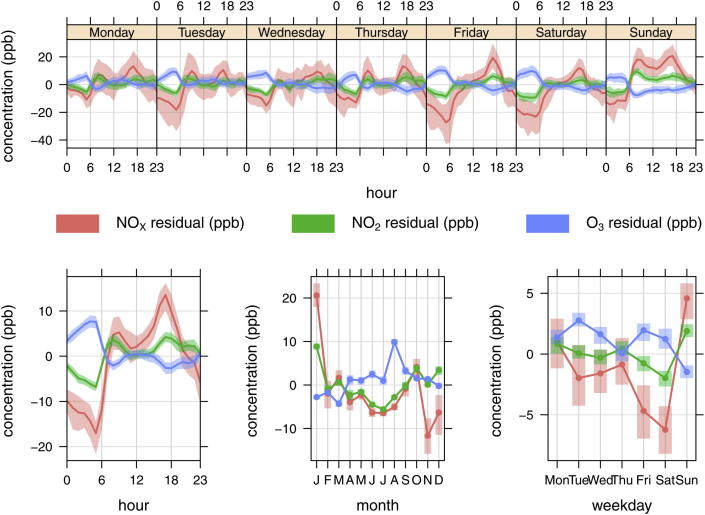
NO_*X*_, NO_2_ and O_3_ residuals (ppb) (modelled–observed) at the Kensington and Chelsea urban background monitoring site. The shaded area represents the 95% confidence interval in the mean.

Assuming that the performance of WRF at Heathrow is reflected widely in London, we have sought to demonstrate the influence of WRF wind speed predictions on NO_*X*_, NO_2_ and O_3_ concentrations at the central London background monitoring site at Kensington and Chelsea. The reason for choosing a background site is that these data are less affected by any bias that local traffic emissions may have. The residual (modelled–observed) concentrations at Kensington and Chelsea are summarised in Fig. 8.

The average hour of daytime series in the bottom left hand corner of Fig. 8 shows that during the morning period in particular there is a negative bias in NO_*X*_ (and NO_2_) concentrations and this has lead to an over prediction of O_3_. The bias in NO_*X*_, NO_2_ and O_3_ are not likely to be linked with the WRF wind speed forecasts, which are well predicted during this period, and these results would suggest that the under prediction of road transport emissions of NO_*X*_ are important at the Kensington monitoring site. The assumption that the CMAQ model bias during the early morning is driven by an under estimate in the NO_*X*_ emissions inventory is further supported by the hour of week plot at the top of Fig. 8, which shows that Friday and Saturday morning in particular, are poorly predicted. Given that WRF wind speed predictions during all morning hours have a similarly small bias (Fig. 7) points to the NO_*X*_ and NO_2_ emissions being the most likely cause. There is a positive bias in the model results during Sundays as well as a tendency to over predict during daytime periods in the remainder of the week (Fig. 8 – top row plot). During the daytime periods a negative bias in WRF predicted wind speed (Fig. 7) is one likely source of the error, and would counteract the effect of under predicting NO_*X*_ emissions. The negative bias in wind speed during the day would also counteract the low emissions of NO_*X*_ at Marylebone Road, discussed in Section 4.1. Finally, the monthly average residuals (bottom row middle plot) suggest that the model under predicts in November and December and over predicts during January for which further investigation is required.

### 4.3 The evaluation of a simple chemical scheme for predicting NO_2_ and O_3_ close to roads in London

At roadside locations in London a simple NO_*X*_–NO_2_–O_3_ chemistry scheme has been used. Despite having been evaluated elsewhere, see Carslaw (2005) and Carslaw and Beevers (2005), we have evaluated the model in 2006 and 2008 using two approaches. First, we have compared the relationship between modelled and measured 2006 NO_2_ and O_3_ concentrations as a function of NO_*X*_ at the Marylebone Road kerbside site and second, we have produced regression plots of the hourly NO_2_ predictions at 16 roadside sites in London, having constrained the model to the hourly NO_*X*_ measurements.

**Fig. 9:**
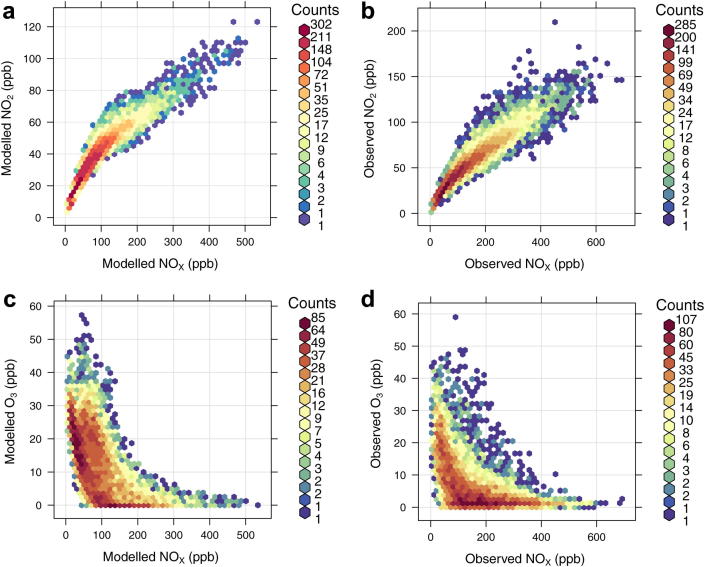
Scatter plots of hourly observed and modelled NO_*X*_ vs. NO_2_ and NO_*X*_ vs. O_3_ (ppb) at the Marylebone Road kerbside site in 2006. The colour scale gives the hourly frequency within each scatter plot to allow clearer interpretation of the results. (For interpretation of the references to colour in this figure legend, the reader is referred to the web version of this article.)

In the first analysis the hourly modelled and observed NO_2_ and O_3_ concentrations have been plotted against NO_*X*_ concentrations and the results summarised in Fig. 9. The comparison between modelled (Fig. 9 (a)) and observed (Fig. 9 (b)) hourly NO_*X*_ and NO_2_ concentrations show that the model tends to under predict both NO_*X*_ and NO_2_ concentrations resulting in smaller concentration ranges. The maximum concentration of NO_*X*_ is ∼500 ppb (model) and 600 ppb (observed) and for NO_2_ is ∼110 ppb (model) and 150 ppb (observed). The model also misses a large group of the highest NO_2_ concentrations (>80 ppb). The under prediction of NO_2_ at Marylebone Road is consistent with the results at other kerbside and roadside sites and with the under prediction of NO_*X*_ and NO_2_ emissions discussed in Section 4.1.

The comparison between modelled (Fig. 9 (c)) and observed (Fig. 9 (d)) hourly NO_*X*_ and O_3_ concentrations shows the model predicts a large number of O_3_ concentrations in the 0–150 ppb NO_*X*_ range and 10–30 ppb O_3_ range, with the equivalent zone of observed concentrations having smaller numbers of hourly values. Furthermore, the zone with the highest number of observed O_3_ concentrations is in the ∼100–400 ppb NO_*X*_ range and within the 0–5 ppb O_3_ range. This high number of low O_3_ concentrations is typical of kerbside locations, but is not replicated by the model which has a smaller number of the lowest O_3_ concentrations.

**Fig. 10:**
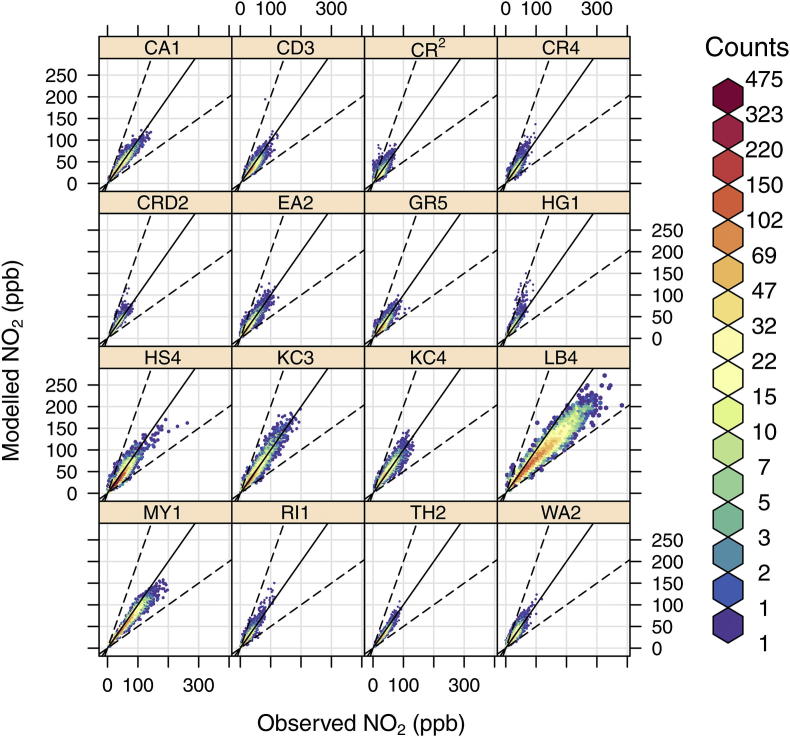
Scatter plots of hourly model vs. measured NO_2_ (ppb) at 16 roadside and kerbside sites in London. The colour scale gives the hourly frequency within each scatter plot to allow clearer interpretation of the results. The black line represents the 1:1 relationship and the dashed lines the 1:2 and 1:0.5 relationships. (For interpretation of the references to colour in this figure legend, the reader is referred to the web version of this article.)

Whilst the comparison between NO_*X*_, NO_2_ and O_3_ for both modelled and observed concentrations is informative it is less helpful in identifying weaknesses in the chemistry model itself. Hence, for the second part of the chemistry scheme evaluation a regression analysis has been undertaken for each hour of 2008 at a subset of 16 London monitoring sites (Table 2), chosen to represent a complete range of roadside concentrations in London. In this case the model was constrained by using the measured hourly NO_*X*_ concentrations at each site and the proportion of directly emitted NO_2_ from the LAEI, removing the uncertainty in predicting NO_*X*_ and further isolating the chemical mechanism. The model vs. observed hourly NO_2_ concentrations are given as scatter plots in Fig. 10 and show good agreement, with the colour scale confirming that whilst scatter exists, the majority of points lie close to the 1:1 line. Regression statistics for the hourly NO_2_ concentrations give *R*^2^ values in the range of 0.81 (Camden 3) to 0.96 (Marylebone Road) and this confirms the good performance of the constrained model across virtually all sites. However, at the Croydon 2 site where the *R*^2^ value is 0.66, the model performs less well although the average NO_2_ concentrations are in close agreement. In contrast, at the Lambeth 4 site the model under predicts observations by 20%. However, Lambeth 4 sits 1–2 m from the kerb on a road with a frequent bus service, all of which have been fitted with regenerating particle traps and emit a large proportion of NO_*X*_ as NO_2_ (Carslaw and Beevers, 2005), making this a difficult location to predict NO_2_. In conclusion, by isolating the NO_*X*_–NO_2_–O_3_ chemical scheme, we have demonstrated that the model compares well with roadside observations in London, despite its simplicity.

## 5 Double counting

**Fig. 11:**
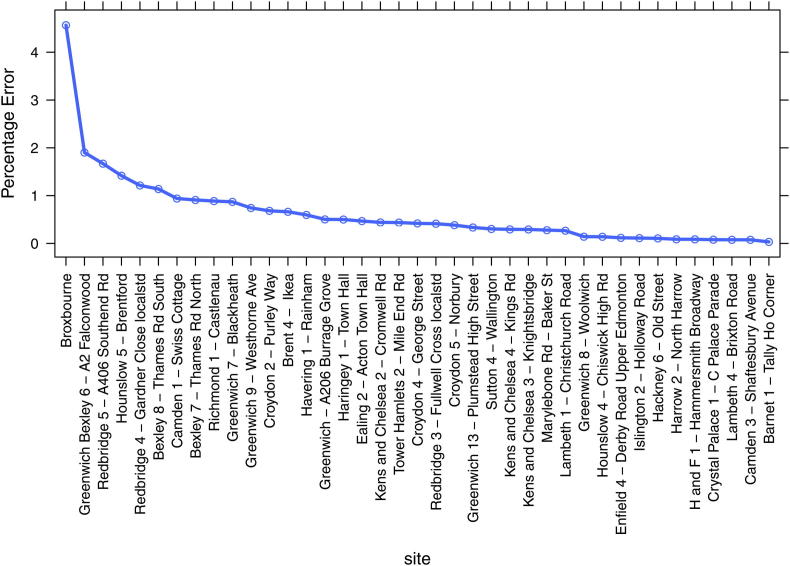
The percentage error in the CMAQ-urban model associated with double counting NO_*X*_ emissions at 39 London roadside sites.

As a consequence of coupling the CMAQ and ADMS models a degree of double counting exists. The reason for this is that the CMAQ model, used for the background concentration estimates, includes all of the emissions sources in London and superimposed upon this background is the ADMS predicted concentrations, which use emissions from the major roads in London. Hence we are effectively counting the major road emissions twice. However, we have demonstrated that this has limited influence on the model predictions, by first, limiting the spatial domain of the roadside model to 225 m from each road source. The 225 m limit in this case is taken to be the limit at which one can measure the annual average influence of a single road above background concentrations and beyond 225 m of a road, the CMAQ concentrations remain unchanged. However, it is accepted that the applicability of the 225 m limit will depend on atmospheric stability and for some hours of the year the local influence of major roads will extend well beyond this limit. Second, whist we accept that some double counting still exists close to roads, it can be tested in a straight forward way. To do this we have used the NO_*X*_ emissions and observations at 39 roadside and kerbside sites closest to the largest roads in London. For each measurement site the 2006 NO_*X*_ emissions and length of road was taken from the LAEI. Where a single road was >3 km long in the LAEI the length was limited to 3 km to be consistent with the size of the CMAQ grids. Using the LAEI estimate, an equal mass of NO_*X*_ emissions was released into a volume source of dimensions 3 km × 3 km × 14 m, equivalent to the lowest layer in the CMAQ model, and the volume source average concentration predicted using the ADMS volume source model (v4.1). The volume average concentration, which represents the magnitude of the double counting effect, was then expressed as a percentage of the roadside NO_*X*_ measurements and the results summarised in Fig. 11.

The results of double counting show a small range at most of the roadside locations with the median percentage error of 0.42% across all sites. This represents a very small error and of little importance in predicting roadside concentrations. One site (Broxbourne) has a larger percentage error of 4.5%, and this is a site outside of the M25 motorway, one of the largest traffic sources in the UK. The site is also at ∼60 m from the centre of the motorway and so sits between a roadside and background site location. At this location the M25 motorway passes through an area of relatively low NO_*X*_ emissions, from other sources, and as such the site represents a worst case location for double counting. Despite the worst case location the error of 4.5% is approximately half the magnitude of the measurement error for NO_*X*_.

## 6 Conclusions

A coupled CMAQ and ADMS modelling system, CMAQ-urban, has been developed for London to give fine temporal and spatial scale (hourly and 20 m × 20 m) NO_*X*_, NO_2_ and O_3_ concentrations. The model used a combination CMAQ v4.6 to predict background concentrations and the ADMS roads model (v2.3) to represent the dispersion from road traffic. Highly detailed road traffic emissions were taken from the LAEI 2006 and 2008 and the model evaluated against 80 measurement sites in London. The majority of tests were undertaken for 2006, although some results for 2008 are presented to support the model evaluation.

The model was able to capture the spatial heterogeneity of NO_2_ and O_3_ concentrations across London. Using observations from 80 monitoring sites at locations which included urban background, suburban, roadside and kerbside sites, the model predicted NO_2_ and O_3_ concentrations reasonably well, although at kerbside sites the model under predicted NO_2_ by a large margin and especially at high hourly concentrations.

By using diagnostic tests such as average modelled and measured concentrations by month of the year and hour of the week, for WRF and CMAQ, we have been better able to differentiate the likely source of model error. For example, the under prediction of NO_*X*_ and NO_2_ concentrations at the Kensington and Chelsea urban background sites during the overnight period is likely to be a result of underestimated road traffic NO_*X*_ emissions, which is also reported elsewhere (Carslaw et al., 2011a), whilst the over prediction of NO_*X*_ concentrations during the day at Kensington is a combination of under prediction of wind speed and underestimated road traffic NO_*X*_ emissions.

The contrast in WRF performance across the UK, which are dominated by rural locations, and those in more urban settings such as Heathrow is confusing and is likely to be as a consequence of the effectiveness of the observational nudging approach adopted within the model. The initial aim of nudging was to resolve the (un-nudged) WRF positive bias in overnight wind speed. However, observational nudging proved to be ineffective at the majority of rural sites, where the over prediction of wind speed remained, but was influential at urban sites such as Heathrow. The unintended consequence of using nudging was to artificially create one negative bias (daytime at Heathrow), whilst attempting to solve the overnight positive bias. The performance of WRF is very important if good CMAQ model performance is to be achieved and care should be taken when using nudging techniques with the WRF model.

A comprehensive evaluation of the simple chemistry model used to predict roadside NO_2_ and O_3_ has been reported elsewhere (Carslaw and Beevers, 2005). However, by constraining the model using measured NO_*X*_ concentrations, and effectively isolating the chemical scheme we have shown the model to be able to predict the hourly NO_2_ very well close to roads, despite its simplicity. This is a reflection of the limited time available for other atmospheric components, such as VOC's, to significantly influence concentrations such as O_3_ and also reflects the modest level of photochemistry experienced in the UK. Hence care should be taken when applying the model to a range of climates.

The influence of double counting was also tested comprehensively across 39 roadside and kerbside sites and for the vast majority of sites this effect is very small. The median double counting error represents 0.42% of the roadside concentration of NO_*X*_. At a ‘worst case’ location close to one of the UK's largest motorways the error of 4.5% of roadside concentrations is still half of the measurement error of NO_*X*_. This is an important finding as it confirms that the one way coupling method described is reasonable, simplifies the combination of the local scale and regional scale models and does not influence the workings of the CMAQ model itself.

The double counting error incurred by coupling the two models is also likely to be smaller for other pollutants such as PM, given that the addition of PM_10_ exhaust and PM_10_ non-exhaust emissions are approximately 5–10% of NO_*X*_. In addition, by coupling the models, use can be made of larger grid sizes without loss of predictive capability. For example, approximately the same results of NO_*X*_, NO_2_ and O_3_ concentrations in London can be obtained using a CMAQ grid of 9 × 9 km, instead of 3 × 3 km, which has the potential to reduce the double counting error by up to a factor of 9 as well as reducing the CMAQ run times.

Any number of additional reasons could be made for the errors occurring at locations close to road traffic, such as the effect of multi-lane roads and the existence of tidal traffic flows, the simplicity of ADMS roads model and the street canyon model within it, as well as defining street canyon characteristics, all of which are potential sources of error. These have not been investigated in this paper and will be the subject of future analysis.

Despite the model errors discussed, we have demonstrated that the use of CMAQ-urban for the prediction of O_3_ and NO_*X*_/NO_2_ concentrations in London is promising. And that at the spatial and temporal scale described this model can be used for health impact assessments, epidemiological study, detailed human exposure assessments and to assess compliance with air quality legislation. Finally, forecasts of PM concentrations, using similar methods and version 5 of CMAQ, will be the subject of future work.

## Figures and Tables

**Table 1:** Statistical measures of model performance in 2006 at KS-kerbside sites, RS-roadside sites, SU-suburban sites and UB-urban background sites.

Pollutant	Site	*n*	FAC2	MB (ppb)	NMB	RMSE (%)	*r*
NO_2_	All	80	0.73	−4.7	−0.17	17.7	0.58
O_3_	All	34	0.61	2.8	0.15	12.1	0.64
NO_2_	KS	7	0.64	−19.0	−0.39	38.1	0.58
NO_2_	RS	38	0.75	−5.0	−0.17	16.3	0.56
NO_2_	SU	13	0.71	−0.8	−0.05	10.8	0.58
NO_2_	UB	22	0.74	−2.1	−0.10	12.3	0.55
O_3_	KS	1	0.37	5.6	0.68	11.1	0.50
O_3_	RS	9	0.60	3.0	0.17	11.9	0.59
O_3_	SU	10	0.66	2.7	0.13	12.4	0.65
O_3_	UB	14	0.61	2.6	0.14	12.1	0.64

**Table 2:** List of 16 roadside and kerbside sites used in the evaluation of the street scale NO–NO_2_–O_3_ chemistry model, including the *R*^2^ estimate between modelled and observed NO_2_ in 2008, the average modelled and observed NO_2_ in 2008 and the emissions based estimate of the exhaust NO_2_–NO_*X*_ ratio.

Site code	Site name	Site type	*R*^2^	Observed NO_2_ (ppb)	Modelled NO_2_ (ppb)	Exhaust NO_2_:NO_*X*_
CA1	Camden 1 – Swiss Cottage	Kerbside	0.93	40	39	0.21
CD3	Camden 3 – Shaftesbury Avenue	Roadside	0.81	41	38	0.19
CR2	Croydon 2 – Purley Way	Roadside	0.66	24	24	0.16
CR4	Croydon 4 – George Street	Roadside	0.84	26	26	0.26
CRD2	Kens and Chelsea 2 – Cromwell Rd	Roadside	0.85	35	35	0.19
EA2	Ealing 2 – Acton Town Hall	Roadside	0.85	29	31	0.24
GR5	Greenwich 5 – Trafalgar Road	Roadside	0.83	27	26	0.22
HG1	Haringey 1 – Town Hall	Roadside	0.87	19	21	0.23
HS4	Hounslow 4 – Chiswick High Rd	Roadside	0.89	38	35	0.23
KC3	Kens and Chelsea 3 – Knightsbridge	Roadside	0.92	48	49	0.24
KC4	Kens and Chelsea 4 – Kings Rd	Roadside	0.86	48	50	0.26
LB4	Lambeth 4 – Brixton Road	Kerbside	0.93	113	90	0.25
MY1	Marylebone Rd – Baker St	Kerbside	0.96	61	55	0.20
RI1	Richmond 1 – Castlenau	Roadside	0.89	23	23	0.24
TH2	Tower Hamlets 2 – Mile End Rd	Roadside	0.90	32	29	0.20
WA2	Wandsworth Town hall	Roadside	0.85	25	26	0.21
